# Asymptomatic only at first sight: malaria infection among schoolchildren in highland Rwanda

**DOI:** 10.1186/s12936-016-1606-x

**Published:** 2016-11-14

**Authors:** Kevin C. Sifft, Dominik Geus, Caritas Mukampunga, Jean Claude Mugisha, Felix Habarugira, Kira Fraundorfer, Claude Bayingana, Jules Ndoli, Irenee Umulisa, Corine Karema, George von Samson-Himmelstjerna, Toni Aebischer, Peter Martus, Augustin Sendegeya, Jean Bosco Gahutu, Frank P. Mockenhaupt

**Affiliations:** 1 Institute of Tropical Medicine and International Health, Charité-University Medicine Berlin, Berlin, Germany; 2 University Teaching Hospital of Butare, University of Rwanda, Butare, Rwanda; 3 Institute for Parasitology and Tropical Veterinary Medicine, Freie Universität Berlin, Berlin, Germany; 4 Malaria and Other Parasitic Diseases Division, Rwanda Biomedical Center, Kigali, Rwanda; 5 Swiss Tropical and Public Health Institute, Basel, Switzerland; 6 University of Basel, Basel, Switzerland; 7 Mycotic and Parasitic Agents and Mycobacteria, Department of Infectious Diseases, Robert Koch-Institute, Berlin, Germany; 8 Institute for Clinical Epidemiology and Applied Biometry, University Hospital, Tuebingen, Germany

## Abstract

**Background:**

*Plasmodium* infection and malaria in school children are increasingly recognized as a relevant public health problem, but data on actual prevalence and health consequences are insufficient. The present study from highland southern Rwanda aimed at estimating infection prevalence among children attending school, at identifying associated factors and at assessing the clinical consequences of these infections.

**Methods:**

In a survey including 12 schools in the Huye district of Rwanda, 1089 children aged 6–10 years were clinically and anthropometrically examined, malaria parasites were diagnosed by microscopy and PCR, haemoglobin concentrations were measured, and socio-economic and behavioural parameters as well as medical histories were obtained.

**Results:**

Upon examination, the vast majority of children was asymptomatic (fever 2.7%). *Plasmodium* infection was detected in 22.4% (*Plasmodium falciparum,* 18.8%); 41% of these were submicroscopic. Independent predictors of infection included low altitude, higher age, preceding antimalarial treatment, and absence of electricity or a bicycle in the household. *Plasmodium* infection was associated with anaemia (mean haemoglobin difference of −1.2 g/dL; 95% CI, −0.8 to −1.5 g/dL), fever, underweight, clinically assessed malnutrition and histories of fever, tiredness, weakness, poor appetite, abdominal pain, and vomiting. With the exception of underweight, these conditions were also increased at submicroscopic infection.

**Conclusion:**

Malaria infection is frequent among children attending school in southern highland Rwanda. Although seemingly asymptomatic in the vast majority of cases, infection is associated with a number of non-specific symptoms in the children´s histories, in addition to the impact on anaemia. This argues for improved malaria surveillance and control activities among school children.

## Background

*Plasmodium* infection can cause a wide variety of illnesses, ranging from asymptomatic infection over uncomplicated malaria to severe and complicated disease [1–4]. Owing to the enormous morbidity and mortality of clinical malaria in young children, the epidemiology and consequences of asymptomatic infection in different settings and age-groups has received comparatively little attention. Increasingly, however, the underrated impact of asymptomatic and/or submicroscopic infections is now being recognized [2, 3, 5–10].

Partial immunity against malaria develops during childhood in endemic regions as a result of repeated exposure. Increasing age has consequently been associated with low and submicroscopic parasite density as well as with asymptomatic infection, particularly among school-age children [5, 11–14]. Most of these infections remain undiagnosed and untreated [14, 15], and—in the case of schoolchildren—are not subject of targeted interventions. However, the significance of these asymptomatic *Plasmodium* infections is likely underestimated as they contribute to inflammatory responses, anaemia, cognitive impairment and delayed physical development [5, 9, 10, 15–20]. Moreover, submicroscopic infections tend to persist for longer than symptomatic ones [13, 21], thereby constituting a sizeable transmission reservoir [2, 3, 14]. This has particular importance in areas of low endemicity considering the increase of submicroscopic (and frequently asymptomatic and untreated) infections with declining malaria transmission [2, 3, 11, 21].

In Rwanda, comprehensive malaria control measures since 2005 have been paralleled by 50% or higher declines in malaria morbidity and mortality [22]. In 2010, in the southern highland Huye district, PCR-assessed *Plasmodium* prevalence among rural children under 5 years of age was 16%, the majority of infections being asymptomatic [18]. This area represents a hypo- to meso-endemic setting as seen in many other parts of the country [12]. No data, however, is available as to the epidemiological characteristics and effects of seemingly asymptomatic and/or submicroscopic *Plasmodium* infections among school-age children living in the highlands of Rwanda. Such knowledge, nevertheless, forms the prerequisites for the design of targeted interventions, the reduction of transmission reservoirs, and, ultimately, elimination.

In 2014, children attending primary schools in the largely rural Huye district of highland southern Rwanda were recruited for a study on anthelminthic treatment efficacy. Based on this study population, the present analysis aimed at estimating the prevalence of *Plasmodium* infection including submicroscopic ones, at identifying socio-demographic and -economic as well as behavioural factors associated with infection, and at assessing the clinical manifestation of these infections with particular focus on anaemia and non-specific symptoms.

## Methods

### Study design, setting and population

This observational, cross-sectional study was conducted in October 2014 in the Huye district, Southern Province, Rwanda. Huye district (population 330,000) is located on Rwanda’s central plateau, which is characterized by an average altitude of 1600–1800 m, a mean temperature of 19 °C and a yearly rainfall of approximately 1200 mm with two rainy seasons (October–December; March–May). Malaria transmission in the area is perennial but low with peaks during the rainy seasons. *Plasmodium falciparum* is the predominant species [12, 18, 23].

**Fig. 1:**
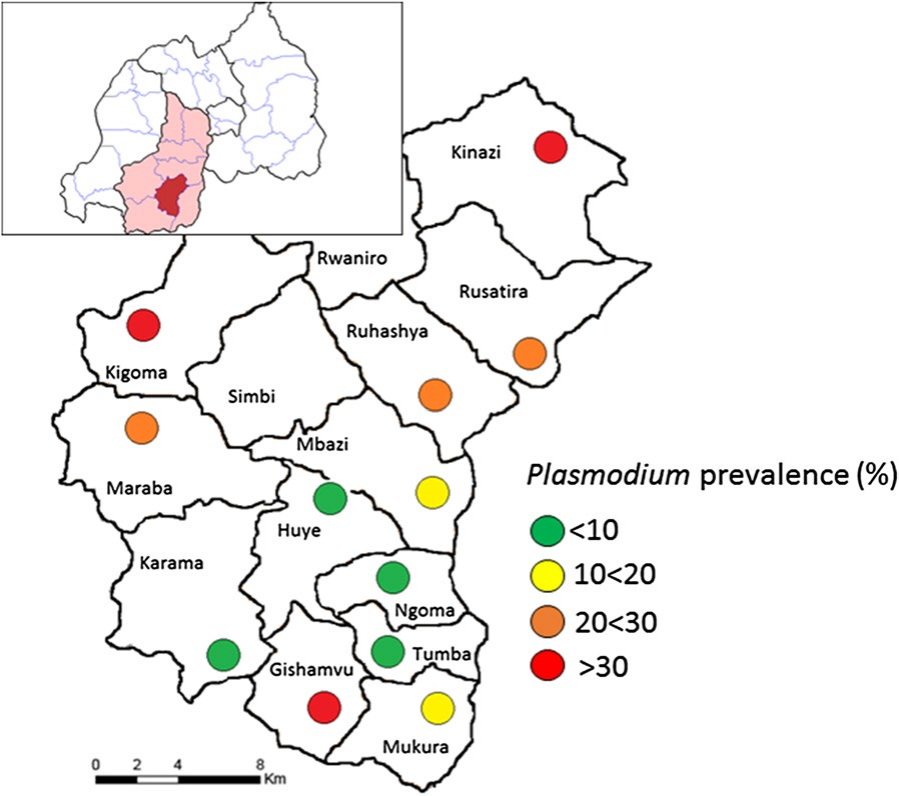
Study area indicating *Plasmodium* prevalence at individual schools. *Map* showing the location of the Southern Province (*pink*) and of Huye district (*red*) in Rwanda as well as the approximate location of schools within Huye sub-districts and the respective prevalence of *Plasmodium* infection. The city of Butare is located in Ngoma sub-district

Huye district is subdivided into 14 sectors (sub-districts; see Fig. 1). For each sector, one primary school of 500–1100 pupils was randomly selected from the district’s school list. Two schools could not be accessed later on, leaving a sample of 12 schools located at altitudes between 1560 and 1850 m. At each school, 150 children aged 6–10 years were randomly selected from the school list aiming at a number of 100 participants per school to be recruited. Several days before examination, meetings were held at each school to explain purpose and procedures of the study to teachers and parents. Permissions were obtained from school directors, heads of local health centres and the district health office. Written informed consent and assent were obtained from the parents and the children, respectively. The study protocol was approved by the Rwandan National Ethics Committee (242/RNEC/2014), and the Rwandan Ministry of Education granted permission to conduct the study (2543/12.00/2014).

### Examinations and laboratory procedures

Alongside routine deworming activities, all participating schools were visited within one week in October 2014. Attending children were clinically examined, and axillary temperature was measured. A brief medical history was obtained from the children, and results were combined with the history as stated by the parents upon interviewing (see below). Age, sex, weight, height, mid-upper arm circumference (MUAC), and fever (axillary temperature ≥37.5 °C) were documented. Weight-for-age z (WAZ) scores and height-for-age z (HAZ) scores were calculated using AnthroPlus [24]. Underweight and stunting were defined as a WAZ or HAZ score < −2 SD, respectively. In addition, clinically assessed malnutrition as determined by the attending physician upon physical examination was documented. Peripheral venous blood was collected into EDTA. Haemoglobin (Hb) concentrations were measured using a HemoCue photometer (Ängelholm, Sweden), and anaemia was defined as a haemoglobin concentration <12 g/dL according to age-related and altitude-adjusted cut-off values [25]. On-site, *P. falciparum* and non-falciparum malaria parasites were detected by immunochromatographic dipstick tests (SD BIOLINE Malaria Ag P.f/Pan, Standard Diagnostics Inc, Gyeonggi-do, Korea); children found positive were treated with artemether–lumefantrine. In case of further illness upon clinical examination, treatment was provided according to Rwandan treatment guidelines, if needed. Blood samples were transported on wet ice to the central laboratory, University Teaching Hospital of Butare (UTBH). Malaria parasites were counted on Giemsa-stained thick blood films per 200 white blood cells (WBCs), and parasite density was calculated assuming a mean WBC count of 8000/µL. A slide was declared negative only after having counted 500 WBCs. Malaria was defined as any microscopically visible parasitaemia *plus* fever or a history of fever within the preceding 48 h. DNA was extracted from stabilized full blood aliquots (AS1 & Qiamp blood kit; Qiagen, Germany), and *Plasmodium* species and submicroscopic infections were identified by semi-nested multiplex polymerase chain reaction (PCR) assays [26]. *Plasmodium* spp. infection was defined as any parasitaemia detected either by microscopy or PCR. Asymptomatic infection herein is defined as the presence of *Plasmodium* spp. in the absence of fever upon examination. This does not exclude the presence of signs and symptoms in the history of the children.

### Socio-economic status and household visits

Trained health workers visited the households of the recruited children and conducted interviews with parents or guardians using a standardized questionnaire. The questionnaire in the local Kinyarwanda language was largely based on precursors used in previous studies in the region [23]. Data collected included information on paternal education and occupation, household characteristics and assets, ability to pay school and insurance fees, ownership and type of livestock, sanitary facilities, water supply and mosquito net ownership and use as well as illnesses or symptoms the child suffered within the preceding 2 weeks, treatment-seeking, and current or past drug use including anti-malarials.

### Statistical analysis

Data from questionnaires, clinical examination, and laboratory results were double-entered into electronic databases and cross-checked. SPSS (SPSS Statistics Version 23.0, IBM Corp., Armonk, NY, USA) was used for statistical data analysis. Geometric mean parasite densities (GMPDs) and 95% confidence intervals (95% CIs) were calculated. Continuous variables were compared between groups by Student’s t test, Mann–Whitney or Kruskal–Wallis tests, and proportions by χ^2^ test or Fisher’s exact test. Odds ratios (ORs) and 95% CIs were computed. Evaluation of determinants of *Plasmodium* infection and of clinical symptoms was performed by simple and multiple logistic regression analysis. Analyses were adjusted for intracluster correlations (clusters  =  schools) by use of generalized estimating equations (GEE, exchangeable correlation structure for subjects within identical villages). Multivariate analysis of factors associated with *Plasmodium* infection was done for the complete sample using multiple imputation for missing data. Stepwise backward selection was performed, and final models included those factors that retained statistical significance. A *P* value <0.05 was considered statistically significant.

## Results

### Study population

Out of 1182 recruited children, *Plasmodium* microscopy and PCR results were available for 1089 children. These form the base of the current analysis, and their characteristics are shown in Table 1. Girls slightly predominated. Upon clinical examination the presence of fever was low (2.7%), and the vast majority of children was clinically unsuspicious; the leading clinical diagnoses were fungal skin infection (8.8%, 95/1076), dental caries (3.4%, 37/1079) and respiratory problems (1.8%, 19/1066). Underweight and/or stunting affected one in six children; anaemia was seen in one in three children. Based on statements of both children and parents, one-third of the children had a history of abdominal pain in the preceding two weeks, followed by cough and fever. One in five children had attended a health facility within the previous 2 weeks and one in six children had received an antimalarial within the preceding month (Table 2).

**Table 1:** Characteristics of 1089 Rwandan schoolchildren

Parameter	Value
No.	1089
Female (%, n/n)	54.1 (589)
Age (years; median, range)	8 (6–10)
Weight (kg; median, range)^a^	21.8 (12.0–41.8)
Height (cm; median, range)^b^	121.5 (99.5–154.0)
Weight-for-age Z-score (WAZ; median, range)^a^	−1.05 (−4.77–2.02)
Underweight (WAZ < 2; %, n/n)	16.0 (173/1080)
Height-for-age Z-score (HAZ; median, range)^b^	−1.06 (−4.99–3.14)
Stunting (HAZ < 2; %, n/n)	17.5 (189/1079)
MUAC (cm; mean ± SD)^a^	16.2 ± 1.3
Clinically assessed malnutrition (%, n/n)	22.0 (238/1080)
Axillary temperature (°C; mean ± SD)	36.5 ± 0.54
Fever (%, n/n)	2.7 (29/1080)
Hb (g/dL; median, range)^a^	12.5 (6.0–16.6)
Anaemia (%, n/n)	36.4 (393/1079)
History in preceding 2 weeks (%, n/n)	
Abdominal pain	34.1 (371)
Cough	28.2 (307)
Fever	25.3 (276)
Poor appetite	19.7 (215)
Weakness	17.2 (178/1032)
Tiredness	13.9 (151)
Diarrhea	12.8 (139)
Vomiting	8.5 (93)
*Plasmodium* infection (%, n/n)	
*Plasmodium* spp. PCR positive	22.4 (244)
*P. falciparum*, PCR positive	18.8 (205)
*P. ovale*, PCR positive	4.9 (53)
*P. malariae*, PCR positive	0.4 (4)
*Plasmodium* spp., microscopy positive^c^	13.9 (151)
Submicroscopic infection	9.3 (101)
Geometric mean parasite density (/µL; 95% CI)	2188 (1582–3026)

^a^ *n* = 1080

^b^ *n* = 1079

^c^Eight children without confirmation by PCR

**Table 2:** Selected socio-economic characteristics of 1019 Rwandan schoolchildren

Parameter	Value
No.	1019
Maternal education (%, n/n)	
None	19.0 (175/923)
Primary	67.2 (620/923)
Secondary or higher; else	11.1 (102/923)
Not ascertainable	2.8 (26/923)
Paternal education (%, n/n)	
None	13.4 (92/685)
Primary	62.0 (425/685)
Secondary or higher; else	13.3 (91/685)
Not ascertainable	11.2 (77/685)
Mother’s occupation farmer or labourer	93.7 (837/932)
Father’s occupation farmer or labourer	93.7 (568/606)
No. of people in household (median, range)^a^	6 (1–14)
No. of siblings (median, range)^a^	3 (0–10)
No. of rooms in household (median, range)^b^	2 (1–9)
Farmland ownership (%, n/n)	27.1 (276/1019)
Household assets (%, n/n)	
Radio	56.9 (577/1014)
Phone	54.9 (556/1013)
Bicycle	16.1 (163/1015)
Electricity	15.8 (161/1017)
Television	7.6 (77/1014)
Fridge	1.9 (19/1014)
Piped water	13.7 (139/1011)
Ability to treat water	37.0 (353/953)
Floor material soil (%, n/n)	75.8 (766/1011)
Unable to pay school fee (%, n/n)	66.5 (614/923)
Unable to pay health insurance (%, n/n)	51.1 (511/1000)
Slept under a bed net last night (%, n/n)	82.7 (842/1018)
Attended health facility within last two weeks (%, n/n)	21.0 (214/1019)
Received anti-malarial treatment within last month (%, n/n)	16.0 (160/1000)
Time to reach next health centre less than 30 min (%, n/n)	25.1 (255/1017)

^a^ *n* = 1014

^b^ *n* = 938

Questionnaire data on SES were available for 1019 participants and reflected a generally poor, rural population (Table 2). Parental primary education predominated but 16% had no formal education at all. The vast majority of children (83%) had slept under a bed net the night before the survey but this proportion declined with age to 78% (168/215) among children aged 10 years (*P* = 0.04 for comparison across years of age).

Infection with *Plasmodium* spp. as detected by PCR was present in more than a fifth of all children and was largely due to *P. falciparum* (Table 1); 41% of these infections were submicroscopic, i.e., below the threshold of microscopy but detected by PCR.

### Factors associated with Plasmodium infection

The prevalence of *Plasmodium* infection ranged from 1.4% in urban Ngoma sector (city of Butare) to 47% in the rural Kigoma sector (*P* < 0.0001; Fig. 1). This impact was considered by GEE in analysing factors associated with *Plasmodium* infection (Table 3). In univariate analysis, several factors were associated with reduced odds of infection including increasing altitude, parental secondary or higher education, various household parameters of comparatively higher SES status (high number of rooms; presence of electricity, non-soil floor material, bicycle, TV set, and mobile phone; ability to pay school fees and health insurance; treatment of water prior to drinking). Sleeping under a bed net the night before examination tended to reduce the odds of infection even though the prevalence of microscopically visible parasitaemia was reduced [19.3% (34/176) vs. 12.8% (108/841), *P* = 0.02]. Positively associated factors were an age of 10 years, stunting, previous health facility attendance or anti-malarial treatment, and a distant health centre. In multivariate analysis, an age of 10 years and antimalarial treatment within the previous month were independent predictors of *Plasmodium* infection whereas increasing altitude, the availability of electricity in the household, and the possession of a bicycle reduced the odds.

**Table 3:** Univariate and multivariate analysis of factors associated with *Plasmodium* infection (PCR) among Rwandan schoolchildren

Variable	No.	Proportion infected (%, n)	Univariate analysis			Multivariate analysis		
			OR	95% CI	*P*	aOR	95% CI	*P*
Age (years)								
6	132	20.5 (27)	1			1		
7	243	17.7 (43)	0.84	0.61–1.15	0.28	0.85	0.64–1.12	0.25
8	268	20.1 (54)	0.98	0.74–1.31	0.90	0.88	0.66–1.18	0.40
9	214	22.0 (47)	1.09	0.78–1.53	0.60	1.10	0.80–1.49	0.56
10	232	31.5 (73)	1.79	1.24–2.57	0.002	1.71	1.15–2.55	0.008
Stunting (HAZ < 2 SD)								
No	890	20.6 (183)	1					
Yes	189	31.2 (59)	1.75	1.22–2.52	0.002	–	–	n.s.
Residence altitude (m)								
<1700	410	33.7 (138)	1			1		
1700–1750	514	19.3 (99)	0.47	0.24–0.93	0.0001	0.61	0.37–1.02	0.061
>1750	165	4.2 (7)	0.09	0.03–0.23	0.031	0.13	0.07–0.23	<0.0001
Maternal education								
None	175	25.9 (47)	1					
Primary	620	22.9 (142)	0.81	0.44–1.50	0.50	–	–	n.s.
Secondary/tertiary	102	8.8 (9)	0.26	0.12–0.61	0.002	–	–	n.s.
Paternal education								
None	92	19.6 (18)	1					
Primary	425	22.4 (95)	1.18	0.67–2.09	0.56	–	–	n.s.
Secondary/tertiary	91	8.8 (9)	0.4	0.16–0.97	0.043	–	–	n.s.
No. of rooms in household								
1–2	575	22.4 (129)	1					
3–4	333	26.1 (87)	1.22	0.79–1.90	0.37	–	–	n.s.
>4	30	3.3 (1)	0.12	0.03–0.50	0.004	–	–	n.s.
Electricity in household								
No	856	25.8 (221)	1			1		
Yes	161	5.6 (9)	0.17	0.06–0.50	0.001	0.31	0.14–0.69	0.004
Floor material								
Plain soil	766	26.8 (205)	1					
Else	245	10.2 (25)	0.31	0.18–0.55	0.0001	–	–	n.s.
Radio in household								
No	437	24.9 (109)	1					
Yes	577	20.6 (119)	0.78	0.55–1.11	0.167	–	–	n.s.
Bicycle in household								
No	852	24.3 (207)	1			1		
Yes	163	13.5 (22)	0.49	0.30–0.79	0.004	0.51	0.30–0.86	0.012
TV set in household								
No	937	24.1 (226)	1					
Yes	77	2.6 (2)	0.08	0.03–0.26	0.0001	–	–	n.s.
Mobile phone in household								
No	457	28.0 (128)	1					
Yes	556	18.0 (100)	0.56	0.35–0.91	0.018	–	–	n.s.
Ability to pay school fees								
No	614	26.1 (160)	1					
Yes	309	14.9 (46)	0.50	0.30–0.81	0.006	–	–	n.s.
Ability to pay health insurance								
No	511	28.4 (145)	1					
Yes	489	16.6 (81)	0.50	0.33–0.77	0.002	–	–	n.s.
Water treatment								
No	600	26.5 (159)	1					
Yes	353	14.4 (51)	0.47	0.29–0.77	0.003	–	–	n.s.
Child slept under bed net last night								
No	176	30.1 (53)	1					
Yes	842	20.9 (176)	0.61	0.35–1.07	0.084	–	–	n.s.
Child attended health facility within preceding 2 weeks								
No	805	20.6 (166)	1					
Yes	214	31.8 (68)	1.79	1.42–2.26	0.0001	–	–	n.s.
Child received any malaria treatment within preceding month								
No	840	17.6 (148)	1			1		
Yes	160	50.0 (80)	4.68	2.67–8.19	0.0001	3.07	1.93–4.89	<0.0001
Time to reach next health centre (min)								
<30	255	14.9 (38)	1					
>30	762	25.3 (193)	1.94	0.95–3.93	0.067	–	–	n.s.

OR, odds ratio; 95% CI, 95% confidence interval; aOR, adjusted odds ratio, adjusted for all other factors shown in table and taking into account cluster effects

Concurrent infection with soil-transmitted helminths (virtually all *Ascaris lumbricoides*, detected by PCR [27]) was not associated with *Plasmodium* infection: 22.5% (72/320) and 20.7% (124/600) of *A. lumbricoides* infected and non-infected children, respectively, harboured *Plasmodium* parasites (*P* = 0.52).

### Clinical manifestations of Plasmodium infection

The vast majority of all children was asymptomatic upon clinical examination even though abdominal pain, cough, fever and other non-specific signs and symptoms were rather common in their histories (see above and Table 1). The influence of *Plasmodium* infection on signs and symptoms was analysed, further separating into microscopic and submicroscopic infections (Table 4). *Plasmodium* infection *per se* was associated with anaemia (mean Hb difference of −1.2 g/dL), fever (though in 7.5% only of those infected), underweight, clinically assessed malnutrition and histories of fever, tiredness, weakness, poor appetite, abdominal pain, and vomiting. At microscopically visible parasitaemia, the presence of these signs and symptoms was generally increased. At submicroscopic infection, anaemia prevalence was almost doubled as compared to uninfected children (mean Hb difference of −0.9 g/dL) but current fever was not increased, and no association with nutritional status was present. Still, among children with submicroscopic infections, fever, tiredness, weakness, poor appetite, and vomiting were significantly more frequently reported to have been present within the preceding two weeks than among their uninfected peers. Notably, the proportion of children affected by these conditions did not differ substantially between children with microscopic or submicroscopic infection (Table 4). No association of *Plasmodium* infection with diarrhoea or cough was observed.

**Table 4:** Clinical manifestations of *Plasmodium* spp. infection among 1071 Rwandan schoolchildren

Parameter	Uninfected	Infected (PCR positive)	*P*	Microscopic infection	*P*	Submicroscopic infection	*P*
No.	823	240		148^b^		100	
Anaemia (%)	29.6	60.4	0.0001	64.2	0.0001	52.0	0.0001
Hb (g/dL; median, range)	12.6 (7.1–16.6)	11.6 (6.0–15.1)	0.0001	11.5 (6.8–15.1)	0.0001	11.9 (6.0–14.5)	0.0001
Current fever (%)	1.0	7.5	0.0001	10.8	0.0001	2.0	0.30
Ax. Temperature (°C; mean ± SD)	36.4 ± 0.4	36.6 ± 0.8	0.0001	36.7 ± 0.9	0.0001	36.5 ± 0.5	0.059
WAZ (SD; median, range)	−0.98 (−3.87–2.02)	−1.25 (−4.51–2.02)	0.001	−1.40 (−4.51–2.02)	0.0001	−1.00 (−3.44–0.42)	0.93
Underweight (WAZ < −2 SD, %)	14.1	20.8	0.045	27.0	0.0001	14.0	0.92
MUAC (cm; mean ± SD)^c^	16.3 ± 1.3	15.9 ± 1.1	<0.0001	15.9 ± 1.2	0.0002	15.9 ± 1.0	0.007
Clinically assessed malnutrition (%)	19.8	28.3	0.005	35.1	0.0001	21.0	0.67
History within preceding 2 weeks (%)							
Fever	19.8	45.0	0.0001	46.6	0.0001	41.0	0.0001
Tiredness	10.6	24.2	0.0001	25.0	0.0001	23.0	0.0001
Vomiting	6.6	14.6	0.0001	14.9	0.0001	14.0	0.0001
Abdominal pain	30.3	45.8	0.001	48.6	0.0001	40.0	0.088
Poor appetite	17.6	27.1	0.023	25.0	0.122	30.0	0.006
Weakness^a^	14.8	25.1	0.006	23.9	0.018	26.8	0.003

Comparisons (*P* values) with uninfected group take into account cluster effects

^a^ *n* = 1019

^b^Eight children without confirmation by PCR

^c^ *n* = 925

Separating into mono-infections with *P. falciparum* and *P. ovale*, no significant differences with respect to the clinical manifestation were observed. One notable exception was anaemia, which tended to be more common among *P. falciparum* infected children (62.5%, 115/184) as compared to their *P. ovale* infected peers (45.7%, 19/35; *P* = 0.06).

## Discussion

In this cross-sectional survey among largely afebrile children attending school in southern Rwanda, 22% were infected with *Plasmodium* spp., predominantly *P. falciparum*, and 41% of these infections were submicroscopic. Infection was predicted by older age, low altitude, previous treatment, and absence of electricity or bicycle. Despite its ostensibly asymptomatic presentation, infection was associated with anaemia, underweight, and a number of non-specific symptoms in the children´s histories.

Infection prevalence varied greatly between locations, with a tendency of decline closer to the city of Butare (located in Ngoma sector, Fig. 1). Such variation is considered to reflect multiple and geographically diverse factors including altitude, rainfall, topography, land use, SES and health-seeking behaviour [28]. Among these, altitude was the strongest predictor of infection in the present study. Declining infection prevalence with increasing altitude is a common pattern also in neighbouring countries [8, 28–31]. For example, in Tanzanian children, infection prevalence declined by 5–21% for every 100-m increase in altitude [28, 30]. The extent of this effect even on a small range between 1560 and 1850 m in the present study is remarkable. The higher prevalence of *Plasmodium* infection at lower altitude, i.e., usually at the valley bottoms, is most likely explained by the closer proximity to vector breeding sites in stagnant waters and commonly practiced rice cultivation. In line with that, valley floors in neighbouring Burundi are considered sources of infection at higher altitudes [31]. Of note, the prevalence of *Plasmodium* infection did not show the usual decline with age, i.e., with cumulative exposure to malaria parasites. This suggests that the intensity of *Plasmodium* transmission in the study area is insufficient to build up immune responses during childhood, which prevent infection. Likewise, in neighbouring Tanzania, the age-dependent decline of infection prevalence has been found to vanish at an altitude similar to that of the present study area [28, 30]. In a previous survey in the study area [18], infection prevalence in young community children increased up to the age of 4–5 years and up to a level of 20–25%, i.e., a prevalence that was seen in most of the children in the present study. Children aged 10 years showed a significantly increased infection prevalence. This may reflect their comparatively lower use of bed nets as well as other behavioural characteristics of older children such as increased exposure due to longer staying outside in the evenings or traveling to areas of higher transmission. Similar to other studies from Rwanda [12, 18, 32], various indicators of low SES associated with *Plasmodium* infection but in multivariate analysis, only the protective parameters household availability of electricity and of a bicycle (each present in 16%) remained. While electricity reflects both, increased SES and proximity to rather urban settings [23], the latter may indicate increased access to malaria-related information, improved awareness and increased usage of curative services. Anti-malarial treatment, mostly with artemether-lumefantrine, within the preceding month was paradoxically associated with increased infection prevalence. This observation was also made among children under 5 years of age in the study area [18] and may stem from recurring parasitaemia following treatment. Drug resistance markers associated with reappearing parasitaemia following artemether-lumefantrine treatment are established in the study area [33, 34], but reports on actual treatment failure are absent. No information on the completeness of treatment was collected, i.e., on adherence to the 3-days regimen, but incomplete drug intake, e.g., in the community or after the first dose in a health facility, may have contributed to reappearing parasitaemia. Moreover, persisting gametocytes could have produced positive PCR results. Alternatively, this finding may reflect the aggregation of infections including rapid re-infections in a specifically vulnerable subset of the population [35].

Recent studies on malaria parasitaemia among school-age children have shown a prevalence range of <1 to 64% in East Africa and of 1 to 83% in West Africa [5]. Symptomatic malaria contributes to school absenteeism, impaired cognition and reduced school performance [5, 10, 16, 20]. As for asymptomatic parasitaemia, predominating in the present study, findings are less clear-cut: in Yemen [6] and DR Congo [36], no influence on children´s cognition was observed while this was the case in Uganda [10], Mali [16], and Kenya [9]. Overall, asymptomatic parasitaemia is abundant; it accounts for three in four infections detected in community settings [4], and many of those are submicroscopic [2, 3]. While some of these infections become symptomatic, most can remain asymptomatic for several months [37, 38]. Counterintuitively, the proportion of submicroscopic infections on a global scale is inversely correlated to transmission intensity, i.e., the proportion is highest at low endemicity [2, 3, 11, 21]. Asymptomatic carriers to a sizeable extent contribute to malaria transmission, also in low endemicity settings [2, 3, 7], even though the magnitude of this impact is not well established [39]. In the present study, more than 90% of *Plasmodium* infected children attending school were considered asymptomatic based on the absence of fever. Nevertheless, anaemia prevalence was doubled in infected children and mean Hb reduced by 1 g/dL. This degree of Hb reduction is approximately half of that observed among younger and also largely asymptomatic children below 5 years in the study area [18]. In addition, infected children more frequently had underweight and clinically assessed malnutrition than their non-infected peers. These findings argue against asymptomatic presentation but rather for a less obvious one. This is even more the case when considering the more frequently positive histories in infected as compared to non-infected children of fever, tiredness, weakness, poor appetite, abdominal pain, and vomiting. The influence of submicroscopic infections on the latter was similar to the one of microscopically visible parasitaemia but the effect on Hb levels was smaller and no association with underweight was observed. These findings add to the notion that “asymptomatic” *Plasmodium* infections do have subtle health consequences and should be regarded as potentially harmful [15]. In fact, symptomless children infected with malaria parasites have been shown to have low-grade inflammation as well as evidence of platelet and endothelial activation, in addition to reduced Hb levels [15].

The present study has limitations. The definition of asymptomatic was based on a single-time point assessment; potentially, some children were in a pre-symptomatic period of parasitaemia at the moment of examination. Likewise, PCR detection of malaria parasites may include gametocytes, e.g., persisting after treatment. Submicroscopic parasitaemia may reflect both, very early and thus asymptomatic infections, which later on could develop to clinical malaria, and low level infections persisting in individuals able to control parasitaemia. Of note, studies in murine models have demonstrated that plasmodial DNA is rapidly removed from circulation after treatment or inoculation of killed parasites [40]. Due to its design as a deworming study, questionnaire-based interviews focused on mainly general and gastrointestinal signs and symptoms whereas more intuitive malaria-related conditions, e.g., shivering or sweats, were not included. Although unfortunate, pronounced symptoms in this regard would likely have been detected upon clinical examination. Moreover, this circumstance enabled detecting significant associations of *Plasmodium* infection with less obvious “soft” symptoms. *Plasmodium* and *Ascaris* infections were not correlated and adjusting for the presence of the latter did not substantially affect the odds of symptoms due to the former. Infections with different *Plasmodium* species were combined since small numbers of non-falciparum malaria infections rendered separate analysis unsubstantial. In clinical terms, *P. falciparum* and *P. ovale* did not differ much, with the exception of a larger contribution to anaemia of the former.

The health and educational consequences of malaria and asymptomatic parasitaemia in school children as well as the role of this large group as a silent reservoir of infection are increasingly recognized [5, 9–20]. School children are not specifically targeted in malaria control programmes so far, and due to the predominance of seemingly asymptomatic parasitaemia, these infections usually remain untreated. The use of insecticide-treated nets (ITNs) in school children is notoriously low [5, 41] but schools are ideally suited to provide education on malaria, prevention and ITNs. This may include information on the importance of adhering to the 3-days regimen of artemisinin-based combination treatment. Schools also can serve as hubs for ITN distribution, and boarding schools in endemic areas should equip their dormitories with ITNs. Chemoprophylaxis or intermittent preventive treatment (IPT) have shown to reduce malaria-associated morbidity in school children [42, 43]. IPT, also termed intermittent parasite clearance in schools (IPCs) or seasonal malaria chemoprevention (SMC), needs to account for the epidemiological characteristics of malaria in a given area, and can greatly reduce the proportion of *Plasmodium* infected children (reviewed in Ref. [5]), and also improve sustained attention [9]. School-based intermittent screening and treatment of malaria is another option but results have been disappointing so far [44]. It remains to be elucidated, which drug-based regimen fits best and is most cost-effective for the different transmission settings across SSA. Integration with other school-based health promotion activities, e.g., routine deworming, is also desirable.

## Conclusion

Morbidity due to *Plasmodium* infection even if asymptomatic at first sight, associated impaired educational performance [5, 10, 16, 20] and contribution to transmission [2, 3, 7] argue for the inclusion of school children into improved surveillance and control activities.
